# Treatment of Insomnia in Multimorbid Elderly

**DOI:** 10.1192/j.eurpsy.2022.153

**Published:** 2022-09-01

**Authors:** G. Stoppe

**Affiliations:** University of Basel, And Mentage, Basel, Switzerland

**Keywords:** nocturnal myoclonus, Benzodiazepines, sleep, pain

## Abstract

The treatment of sleep disorders in older people requires knowledge of the changes in sleep in old age. In the case of multimorbid older people, pharmacological aspects such as interactions must also be taken into account. Sleep in old age is characterised by a lower depth of sleep and more frequent awakenings. The duration of sleep corresponds to that in middle adulthood. In multimorbid older people, sleep is often chronically impaired by pain and/or obstructive breathing disorders. Many medications can have a negative effect on sleep. This applies to cortisone, for example. Antipsychotics can also worsen sleep by worsening nocturnal myoclonia. Ideally, sleep disorders should first be addressed non-pharmacologically. For benzodiazepines, preparations with a short half-life should be chosen. An algorithm is presented. References:

Gulia KK, Kumar VM. Sleep disorders in the elderly: a growing challenge. Psychogeriatrics 2018;18(3):155-165. Samara MT, Huhn M, Chiocchia V, Schneider-Thoma J, Wiegand M, Salanti G, Leucht S. Efficacy, acceptability, and tolerability of all available treatments for insomnia in the elderly: a systematic review and network meta-analysis. ActaPsychiatr Scand. 2020;142(1):6-17.

**Disclosure:**

No significant relationships.

